# Supplementary material for: A Plasmodium falciparum genetic cross reveals the contributions of pfcrt and plasmepsin II/III to piperaquine drug resistance
Source: bioRxiv. 2023 Sep 17:2023.06.06.543862. Preprint. [Version 2] doi: 10.1101/2023.06.06.543862 (PMC10515748; doi:10.1101/2023.06.06.543862)
Supplement: 1 [file NIHPP2023.06.06.543862V2-supplement-1.pdf]

bioRxiv preprint doi: https://doi.org/10.1101/2023.06.06.543862; this version posted September 17, 2023. The copyright holder for this preprint (which was not certified by peer review) is the author/funder, who has granted bioRxiv a license to display the preprint in perpetuity. It is made available under aCC-BY 4.0 International license.

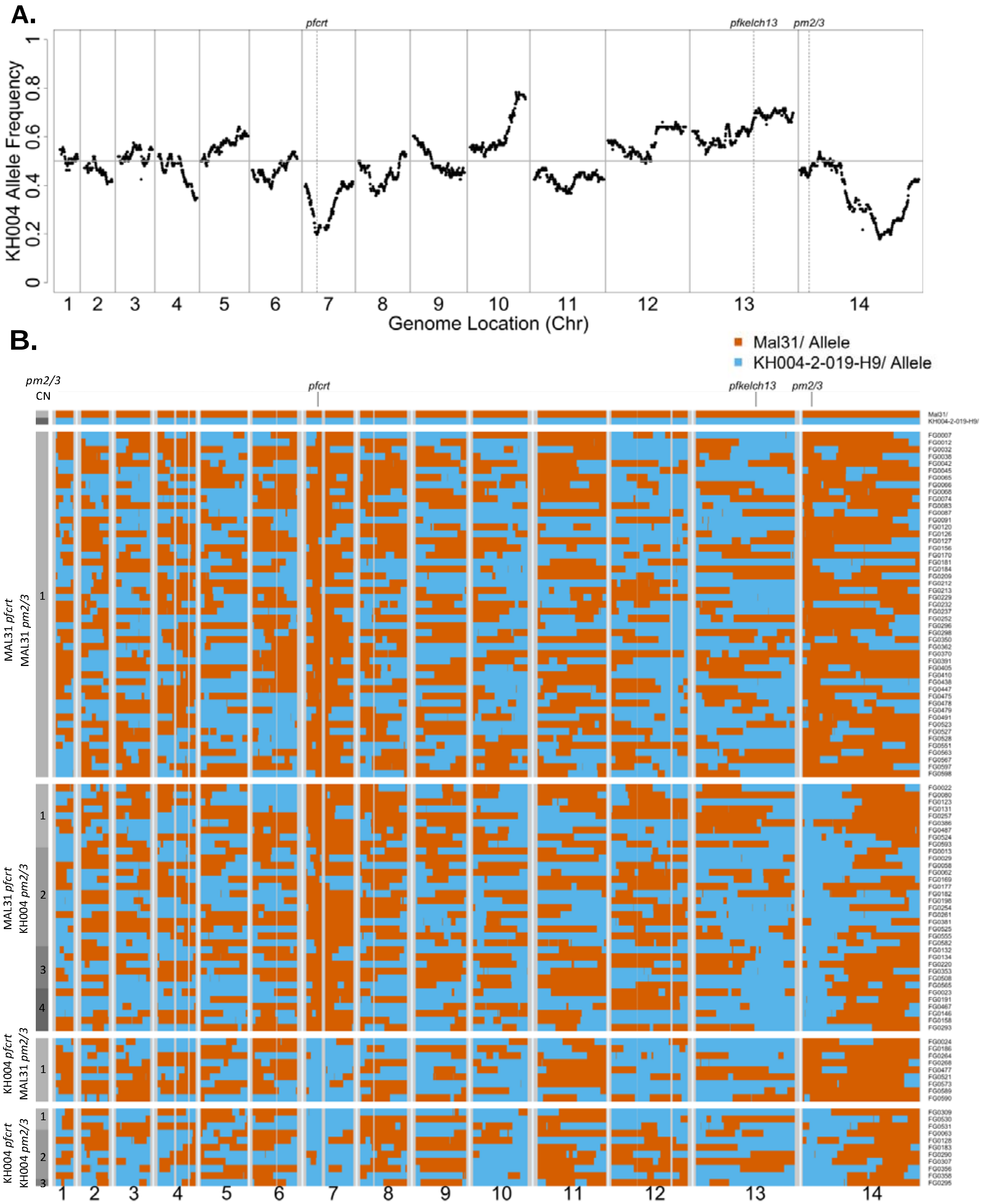

Figure S1: Inheritance breakdown of KH004xMal31 genetic cross. *A) From this cross, we see two strong skews in inheritance on chromosomes 7 and 14, both favoring the Mal31 allele. This selective inheritance has previously been observed in other genetic crosses between Southeast Asian and African parasites. The chromosome 7 peak is centered around pfcrt and therefore potentially represents a fitness cost associated with the inheritance of the KH004 allele at these positions. B) Aside from the selective inheritance on chromosomes 7 and 14, we observe no significant co-inheritance patterns between pfcrt, pm2/3, or kelch13. Inheritance of the KH004 allele is denoted in blue and the Mal31 allele in orange.*

bioRxiv preprint doi: https://doi.org/10.1101/2023.06.06.543862; this version posted September 17, 2023. The copyright holder for this preprint (which was not certified by peer review) is the author/funder, who has granted bioRxiv a license to display the preprint in perpetuity. It is made available under aCC-BY 4.0 International license.

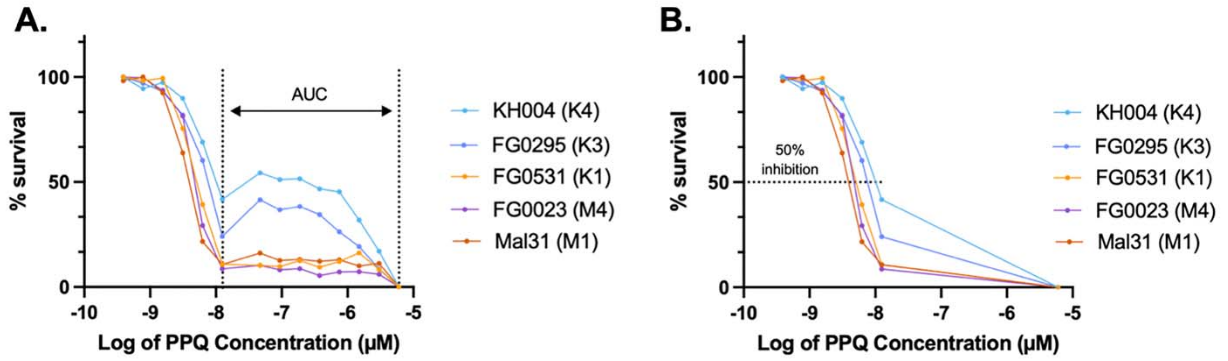

Figure S2: Shape of dose-response curve changes based on pfcrt and pm2/3 genotype. *Traditional IC_{50} measurements have been a challenge with PPQ due to the biphasic dose-response curve observed in PPQ-R parasites. A) We have found inheritance of the KH004 pfcrt allele and multiple copies of pm2/3 are required for producing a biphasic curve. Progeny inheriting the Mal31 pfcrt allele produce do not produce a biphasic curve, regardless of CNV. Additionally, all parasites expressing a single copy of pm2/3 produce a sigmoidal curve. To account for these various dose-response curve shapes, AUC is used as a measure of resistance, with a greater AUC indicating decreased PPQ susceptibility. B) By using a limited number of points in our dose response curve (LP-IC_{50}), we can eliminate the secondary peak in the curve and measure an IC50 independent of original curve shape.*

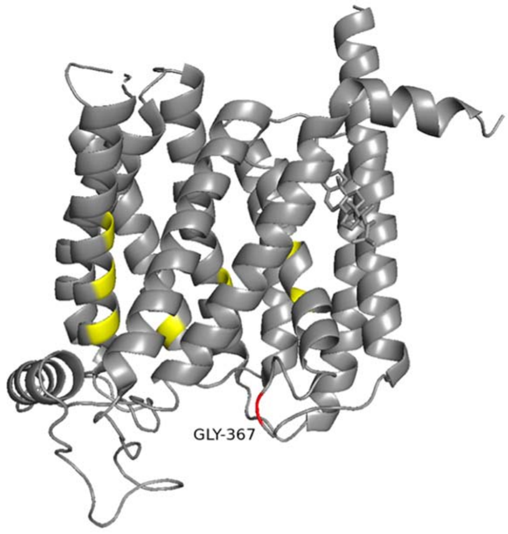

Figure S3: Structural position of G367C Substitution: *A) Alphafold model of Dd2 PfCRT with previously documented in vitro PPQ-R associated mutations identified (yellow). Additional highlighted AA substitutions and the novel G367C substitution carried by KH004 (red).*

bioRxiv preprint doi: https://doi.org/10.1101/2023.06.06.543862; this version posted September 17, 2023. The copyright holder for this preprint (which was not certified by peer review) is the author/funder, who has granted bioRxiv a license to display the preprint in perpetuity. It is made available under aCC-BY 4.0 International license.

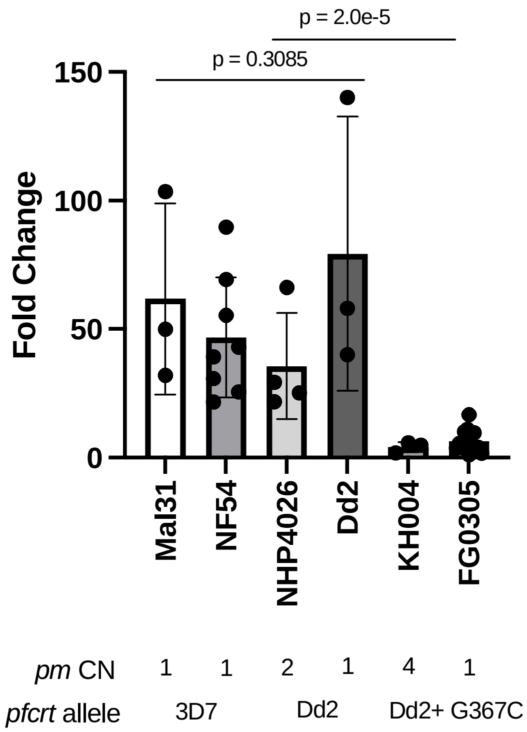

Figure S4: PfCRT G367C directly confers PPQ-resistance *PSA between KH004, FG0305 (selfed KH004 with single copy pm) and two other southeast Asian parasites (Dd2 and NH4026) which carry a Dd2-like PfCRT allele, and two African parasites carrying a WT PfCRT identifies that a Dd2 PfCRT background alone is not sufficient for PPQ-R. This further supports the conclusion that PfCRT G367C directly confers PPQ resistance.*

734

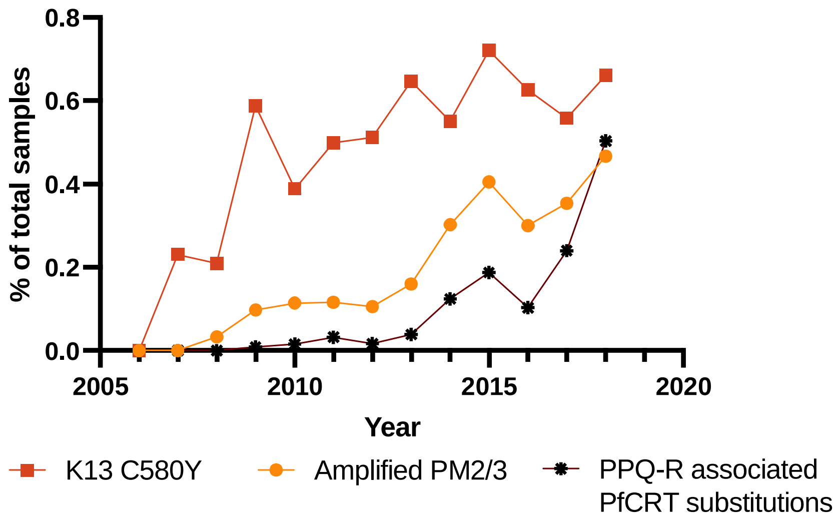

Figure S5: Rise of pm2/3 amplification and novel pfcrt mutations associated with PPQ-R in the GMS. *Using available data from MalariaGEN Pf7 release (n = 3,359), samples from Cambodia, Vietnam, and Laos were analyzed for the association between pm2/3 amplification and novel PPQ-R conferring pfcrt mutations. The rise in novel pfcrt PPQ-R mutations immediately following amplification of pm2/3 further confirms the important roles of both pm2/3 and pfcrt in the evolution of PPQ-R.*

bioRxiv preprint doi: https://doi.org/10.1101/2023.06.06.543862; this version posted September 17, 2023. The copyright holder for this preprint (which was not certified by peer review) is the author/funder, who has granted bioRxiv a license to display the preprint in perpetuity. It is made available under aCC-BY 4.0 International license.

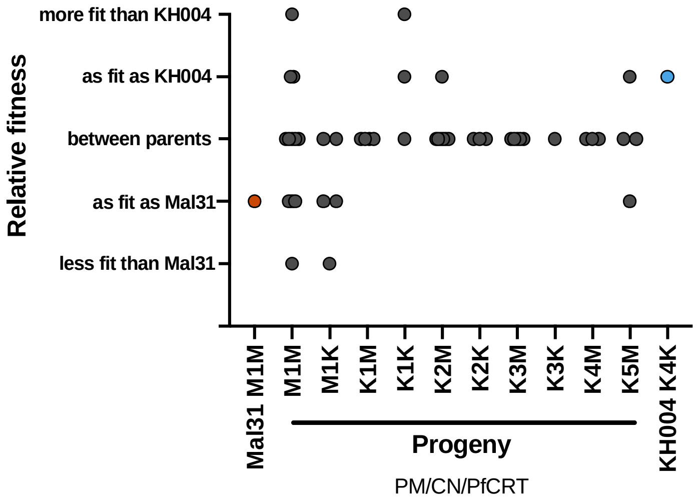

Figure S6: Competitive growth assessment between progeny and parents. *Head-to-head competitive growth assays were set up between progeny and parents and were ranked based on competitive outcome. The fitness level of most progeny fell in between the two parents and no apparent clustering was observed between relative fitness and pfcrt or pm2/3 genotype.*
